# GPT-3.5 Turbo and GPT-4 Turbo in Title and Abstract Screening for Systematic Reviews

**DOI:** 10.2196/64682

**Published:** 2025-03-12

**Authors:** Takehiko Oami, Yohei Okada, Taka-aki Nakada

**Affiliations:** 1 Department of Emergency and Critical Care Medicine, Chiba University Graduate School of Medicine, 1-8-1 Inohana, Chuo, Chiba, 260-8677, Japan, 81 432262372; 2 Department of Preventive Services, Kyoto University Graduate School of Medicine, Kyoto, Japan; 3 Health Services and Systems Research, Duke-NUS Medical School, National University of Singapore, Singapore, Singapore

**Keywords:** large language models, citation screening, systematic review, clinical practice guidelines, artificial intelligence, sepsis, AI, review, GPT, screening, citations, critical care, Japan, Japanese, accuracy, efficiency, reliability, LLM

## Abstract

This study demonstrated that while GPT-4 Turbo had superior specificity when compared to GPT-3.5 Turbo (0.98 vs 0.51), as well as comparable sensitivity (0.85 vs 0.83), GPT-3.5 Turbo processed 100 studies faster (0.9 min vs 1.6 min) in citation screening for systematic reviews, suggesting that GPT-4 Turbo may be more suitable due to its higher specificity and highlighting the potential of large language models in optimizing literature selection.

## Introduction

Systematic reviews are essential in guideline development. Manual citation screening, however, is a time-consuming and labor-intensive process, often resulting in human errors and increased workloads [1,2]. Large language models (LLMs) have demonstrated the ability to comprehend and process natural language, underscoring their utility in medical applications [3]. Consequently, LLMs have emerged as promising tools for citation screening in systematic reviews [4].

LLMs, including GPT, Gemini, and Claude, could serve as secondary reviewers in title and abstract screening, with the downsides of needing to reconcile false positives and potentially missing some relevant citations [5-8]. Although more advanced LLMs are expected to outperform previous models in sensitivity, specificity, and efficiency [9], the full impact of model development in citation screening remains to be fully understood.

This study aimed to compare accuracy and efficiency between GPT-3.5 Turbo and GPT-4 Turbo (OpenAI)—widely used LLMs in the medical field—in title and abstract screening.

## Methods

We conducted a post hoc analysis of our previous study to evaluate the performance of GPT-3.5 Turbo and GPT-4 Turbo in LLM-assisted title and abstract screening, using data from 5 clinical questions (CQs) developed for the Japanese Clinical Practice Guidelines for Management of Sepsis and Septic Shock 2024 [6,10]. The two models determined the relevance of each reference based on patient characteristics, interventions, comparisons, and study designs specific to the selected CQs (Table S1 in Multimedia Appendix 1). LLM-assisted screening was conducted by using Python (v3.9.0) and the OpenAI application programming interface. The same prompt—optimized to increase sensitivity from our previous study—was applied to both models (Multimedia Appendix 1). Evaluation metrics were expressed as sensitivity and specificity with 95% CIs, using the final list of included studies in the conventional review as the reference standard. These measures were aggregated to estimate the pooled sensitivity and specificity of LLM-assisted procedures. Additionally, we measured the time taken by each model to screen 100 studies. Further analysis details are available in Multimedia Appendix 1. LLM-assisted citation screening was conducted between June 6 and 7, 2024. STARD (Standards for Reporting of Diagnostic Accuracy) guidelines were followed.

## Results

**Figure 1.**
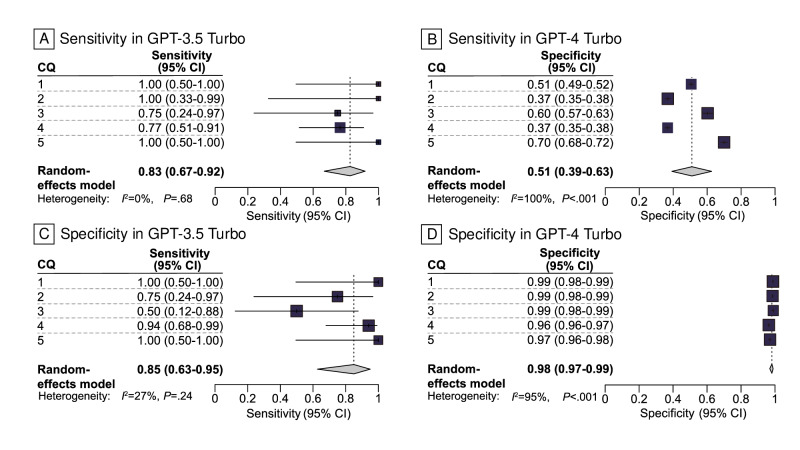
Comparison of GPT-3.5 Turbo’s and GPT-4 Turbo’s accuracy in citation screening. The results of the included publications were qualitatively analyzed, using the conventional method as the standard reference. The individual sensitivity and specificity for each CQ and the integrated sensitivities and specificities across CQs 1 to 5 were compared between GPT-3.5 Turbo (A and B) and GPT-4 Turbo (C and D), with 95% CIs and inconsistency values (*I*^2^). CQ: clinical question.

**Figure 2.**
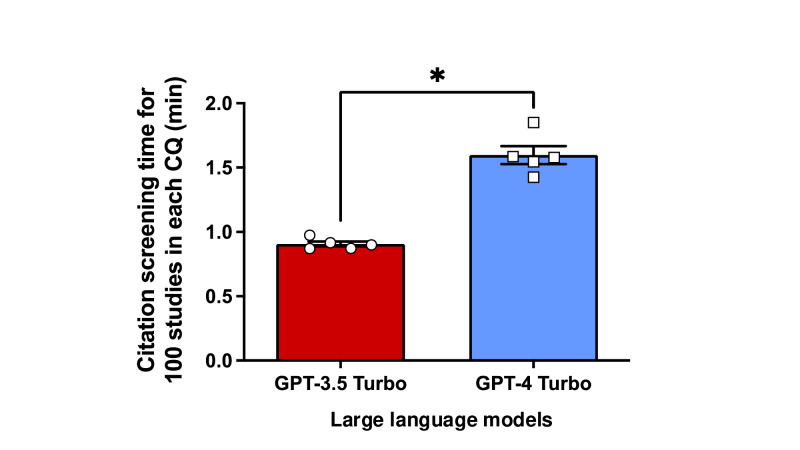
Comparison of citation screening time for 100 studies between GPT-3.5 Turbo and GPT-4 Turbo. The difference in processing time was 0.69 (95% CI 0.53-0.86) min. An unpaired, 2-tailed *t* test was used for analysis. CQ: clinical question. *Statistically significant at *P*<.001.

In the conventional citation screening process, 0.24% (41/16,669) of citations for 5 CQs were selected during the full-text evaluation. GPT-3.5 Turbo exhibited a combined sensitivity and specificity of 0.83 (95% CI 0.67‐0.92) and 0.51 (95% CI 0.39‐0.63), respectively (Figure 1). In contrast, GPT-4 Turbo demonstrated greater performance, with a sensitivity and specificity of 0.85 (95% CI 0.63‐0.95) and 0.98 (95% CI 0.97‐0.99), respectively (Figure 1, Table S2 in Multimedia Appendix 1). A significant difference was found in specificity between both models (median difference 0.48, 95% CI 0.29 to 0.62) but not in sensitivity (median difference −0.06, 95% CI −0.50 to 0.23; Figure S1 in Multimedia Appendix 1). GPT-3.5 Turbo processed 100 studies faster than GPT-4 Turbo (0.9 min vs 1.6 min, respectively; mean difference 0.69, 95% CI 0.53-0.86 min; Figure 2, Table S3 in Multimedia Appendix 1).

## Discussion

Our analysis showed that GPT-4 Turbo had similar sensitivity to but higher specificity than GPT-3.5 Turbo, with minimal impact on screening speed. The high specificity of GPT-4 Turbo is crucial for reducing workloads in subsequent review phases by minimizing the inclusion of irrelevant studies. Although GPT-3.5 Turbo demonstrated shorter screening times, its lower specificity may increase review times. Given the trade-off relationship between sensitivity and specificity, LLM users should choose the optimal model according to their situations.

Our findings emphasize the impact of LLMs’ development on their performance for citation screening and the need to reinforce a model’s suitability for accurate and reliable citation screening [8,9]. Although LLMs are promising tools for title and abstract screening in systematic reviews [4], caution is warranted until further investigations validate their reliability in real-world applications.

This study has several limitations. First, the focus on sepsis limits the generalizability of the findings. Further validation with diverse datasets in other medical domains would enhance the robustness of our conclusions. Second, the post hoc nature of this study may have introduced selection bias. Third, evaluation metrics depend on the reference standard. Fourth, this study did not investigate other LLMs or prompts created via prompt engineering, which could have improved performance. Fifth, the results were based on the LLMs available at the time of analysis. Future investigations should use OpenAI o1 or newer models.

In conclusion, GPT-4 Turbo demonstrated higher specificity than and similar sensitivity to GPT-3.5 Turbo, making GPT-4 Turbo more suitable for systematic reviews, despite having slightly longer processing times.

## Supplementary material

10.2196/64682 Multimedia Appendix 1 Supplementary content regarding the clinical questions, the conventional citation screening, the command prompt used, the automated implementation of the citation screening process, and further data on the comparisons conducted.

## Abbreviations

CQ: clinical question
LLM: large language models
STARD: Standards for Reporting of Diagnostic Accuracy
