# Supplementary material for: GPT-3.5 Turbo and GPT-4 Turbo in Title and Abstract Screening for Systematic Reviews
Source: JMIR Med Inform. 2025 Mar 12;13:e64682. doi: 10.2196/64682 (PMC11922487; doi:10.2196/64682)
Supplement: Multimedia Appendix 1 [file medinform-v13-e64682-s001.docx]

**Multimedia Appendix 1**

**Multimedia Appendix 1**

**Table S1.** List of patients/populations/problems, interventions, and comparisons of selected clinical questions

**Table S2.** Statistics on accuracy of the large language model-assisted citation screening

**Table S3.** Comparison of the citation screening times for 100 studies per person between GPT-3.5 Turbo and GPT-4 Turbo

**Figure S1.** Comparison of the sensitivity and specificity between GPT-3.5 Turbo and GPT-4 Turbo

**Multimedia Appendix 1**

**Table of Contents**

**1. Clinical questions in the Japanese Clinical Practice Guidelines for Management of Sepsis and Septic Shock**

**2. Conventional citation screening**

**3. Command prompt for the large language model-assisted citation screening task**

**4. Automated implementation of citation screening process**

**Multimedia Appendix 1**

**1. Clinical questions in the Japanese Clinical Practice Guidelines for Management of Sepsis and Septic Shock**

The Japanese Clinical Practice Guidelines for the Management of Sepsis and Septic Shock were released in 2020 by the Japanese Society of Intensive Care Medicine (JSICM) and the Japanese Association for Acute Medicine (JAAM), which aimed to introduce a set of guidelines tailored to the specific clinical context of Japan for managing sepsis and septic shock [1]. The updated edition of the J-SSCG 2024 will be released in 2025.

The development of the J-SSCG 2024 involved a thorough review of the relevant literature sourced from CENTRAL, PubMed, and Ichushi-Web, a Japanese biomedical database. This process included a meticulous search strategy covering key studies and was limited to literature in Japanese and English. All identified titles and abstracts were systematically managed using EndNote (Clarivate Analytics) for the systematic review process [2]. A previous report on an automated citation screening software used the same clinical questions [3]. The systematic review process had been completed when the performance of the large language model (LLM)-assisted citation screening was assessed.

**2. Conventional citation screening**

EndNote-processed files were transferred to Rayyan, a web-based tool designed to support systematic reviews [4]. This process involved two reviewers independently assessing the titles and abstracts. Any disagreements were resolved through consensus or a third reviewer's decision. These articles, selected using a manual citation screening method, served as reference standards in this study. Notably, the results from manual screening were kept separate from those of the LLM-assisted screening to maintain the integrity of the evaluation. Moreover, the authors did not conduct a conventional literature search for the clinical questions (CQs) addressed. The time required for conventional screening was measured using the time-tracking functionality built into Rayyan, based on data previously recorded during screening sessions.

**3. Command prompt for the large language model-assisted citation screening task in CQ1**

In the previous study, we have optimized the command prompt to increase sensitivity [5]. The initial prompt was designed based on the inclusion criteria outlined in the CQ framework. At first, the following command prompt was used for the LLM-assisted citation screening in CQ1:

You are conducting a systematic review and meta-analysis, focusing on a specific area of medical research. Your task is to evaluate research studies and determine whether they should be included in your review. To do this, each study must meet the following criteria:

Target Patients: Adult patients (18 years old or older) diagnosed with or suspected of having infection, bacteremia, or sepsis.

Intervention: The study investigates the effects of balanced crystalloid administration.

Comparison: The study compares the above intervention with 0.9% sodium chloride administration.

Study Design: The study must be a randomized controlled trial.

Additionally, any study protocol that meets these criteria should also be included.

However, you should exclude studies in the following cases:

The study does not meet all of the above eligibility criteria.

The study's design is not a randomized controlled trial. Examples of unacceptable designs include case reports, observational studies, systematic reviews, review articles, animal experiments, letters to editors, and textbooks.

After reading the title and abstract of a study, you will decide whether to include or exclude it based on these criteria. Please answer with include or exclude only.

Title: ----------------------

Abstract

-------------------------------------------------------------------------------------------------------

Following the LLM-assisted citation screening using the initial command prompt, the sensitivity was relatively low (0.75). To understand the reasons for the LLM's incorrect judgments, we analyzed false-positive and false-negative results. Our investigation revealed that the LLM strictly adhered to the inclusion criteria outlined in the CQ framework, leading to overly rigid exclusion decisions. To address this issue, we revised the command prompt to make the inclusion more flexible, aiming to improve the sensitivity of LLM-assisted citation screening, as described below. The impact of these modifications on the performance of LLM-assisted citation screening tasks was an increase in sensitivity to 0.89 with specificity largely maintained (0.98).

You are conducting a systematic review and meta-analysis, focusing on a specific area of medical research. Your task is to evaluate research studies and determine whether they should be included in your review. To do this, each study must meet the following criteria:

Target Patients: Adult patients (18 years old or older) diagnosed with or suspected of having infection, bacteremia, or sepsis. If there is a possibility that the study population includes patients with sepsis, the study should be included.

Intervention: The study investigates the effects of balanced crystalloid administration.

Comparison: The study compares the above intervention with 0.9% sodium chloride administration.

Study Design: The study must be a randomized controlled trial.

Additionally, any study protocol that meets these criteria should also be included.

However, you should exclude studies in the following cases:

The study does not meet all of the above eligibility criteria.

The study's design is not a randomized controlled trial. Examples of unacceptable designs include case reports, observational studies, systematic reviews, review articles, animal experiments, letters to editors, and textbooks.

After reading the title and abstract of a study, you will decide whether to include or exclude it based on these criteria. If there is uncertainty in the decision due to a lack of adequate information as you evaluate each domain, you will answer include to minimize the possibility of inadvertently excluding potentially relevant literature. Please answer with include or exclude only.

Title: ----------------------

Abstract

-------------------------------------------------------------------------------------------------------

**4. Automated implementation of citation screening process**

We developed an automated implementation program for the citation screening process, allowing LLMs to independently conduct citation screening using Pandas (v1.0.5) and Python (v3.9.0) through the OpenAI application programming interface, as described in the previous report [5]. This approach involved instructing the LLM to autonomously implement citation screening tasks in line with the patient/population/problem, intervention, comparison, and study design specified in each CQ set by the J-SSCG2024 committee, as manually done in citation screening. After importing the datasets for five CQs used in citation screening, the LLM, without previous knowledge, decided to either include or exclude each citation based on the patient/population/problem, intervention, comparison, and study design of the given CQ. To evaluate the performance, we tracked the time taken to accomplish the task using Python. Analyses were performed using the meta-package in R (version 4.1.2; R Foundation for Statistical Computing) and GraphPad Prism version 10.4.1 (GraphPad Software, Boston, MA). The code for this procedure is available at https://github.com/seveneleven711thanks39/gpt-assisted_citation_screening.git.

**Table S1. List of the patient/population/problem, intervention, and comparison of the selected clinical questions**

|  | Patient, population, problem | Intervention | Comparison |
| --- | --- | --- | --- |
| CQ1 | Adult patients (18 years old or older) diagnosed with or suspected of having infection, bacteremia, or sepsis | Balanced crystalloid administration | 0.9% sodium chloride administration |
| CQ2 | Adult patients (18 years old or older) with sepsis or suspected as sepsis, infection, bacteremia or patients admitted to ICU | Targeting a higher mean arterial pressure | Targeting a lower mean arterial pressure |
| CQ3 | Adult patients (18 years old or older) with sepsis presenting with severe metabolic acidosis or patients admitted to ICU | Sodium bicarbonate administration | No sodium bicarbonate administration |
| CQ4 | Adult patients (18 years old or older) with sepsis or septic shock | Usual care with at least one of the following tissue perfusion parameters: lactate/lactate clearance, capillary refill time, ScvO_2_/SvO_2_, and P(v-a) CO_2_/C (a-v) O_2_. | Usual care with different parameters mentioned in the interventional group or standard care without the utilization of any specific tissue perfusion parameters |
| CQ5 | Adult patients (18 years old or older) with sepsis, sepsis-induced hypotension, or septic shock | Restrictive fluid management aiming to reduce the amount of fluid therapy for up to 24 h | Conventional fluid management or non-restrictive fluid management defined by authors |

CQ: clinical question; ICU: intensive care unit

**Table S2. Statistics on the accuracy of large language model-assisted citation screening**

**A. GPT-3.5 Turbo**

|  | TN | FP | FN | TP |
| --- | --- | --- | --- | --- |
| Primary analysis^a^ | | | | |
| CQ1 | 2,844 | 2,782 | 0 | 8 |
| CQ2 | 1,256 | 2,158 | 0 | 4 |
| CQ3 | 624 | 410 | 1 | 3 |
| CQ4 | 1,581 | 2,728 | 4 | 13 |
| CQ5 | 1,572 | 673 | 0 | 8 |
| Secondary analysis^b^ | | | | |
| CQ1 | 2,837 | 2,685 | 7 | 105 |
| CQ2 | 1,256 | 2,145 | 0 | 17 |
| CQ3 | 624 | 400 | 1 | 13 |
| CQ4 | 1,578 | 2,678 | 7 | 63 |
| CQ5 | 1,571 | 643 | 1 | 38 |

**B. GPT-4 Turbo**

|  | TN | FP | FN | TP |
| --- | --- | --- | --- | --- |
| Primary analysis^a^ | | | | |
| CQ1 | 5,558 | 68 | 0 | 8 |
| CQ2 | 3,366 | 48 | 1 | 3 |
| CQ3 | 1,024 | 10 | 2 | 2 |
| CQ4 | 4,155 | 154 | 1 | 16 |
| CQ5 | 2,181 | 64 | 0 | 8 |
| Secondary analysis^b^ | | | | |
| CQ1 | 5,485 | 37 | 73 | 39 |
| CQ2 | 3,363 | 38 | 5 | 12 |
| CQ3 | 1,020 | 4 | 6 | 8 |
| CQ4 | 4,133 | 123 | 23 | 47 |
| CQ5 | 2,179 | 35 | 2 | 37 |

CQ, clinical question; FN, false negative; FP, false positive; TN, true negative; TP, true positive

^a^ The list of included studies for qualitative analysis using the conventional method was set as the standard reference.

^b^ The list of included studies after title/abstract screening using the conventional method was set as the standard reference.

**Table S3. Comparison of citation screening time for 100 studies between GPT-3.5 Turbo and GPT-4 Turbo**

|  | GPT-3.5 Turbo | GPT-4 Turbo |
| --- | --- | --- |
|  | Time (min) | Time (min) |
| CQ1 | 0.9 | 1.5 |
| CQ2 | 0.9 | 1.6 |
| CQ3 | 0.9 | 1.4 |
| CQ4 | 0.9 | 1.6 |
| CQ5 | 1.0 | 1.9 |

LLM: large language model, CQ: clinical question

**Figure S1. Comparison of the sensitivity and specificity between GPT-3.5 Turbo and GPT-4 Turbo**

**A**

**B**

The difference in sensitivity was −0.06 (95% confidence interval [−0.50 to 0.23], p = 0.60). The difference in specificity was 0.48 (95% confidence interval [0.29 to 0.62], p = 0.008). The asterisk indicates statistically significant. The Mann-Whitney *U* test was used for the analyses.

**eReferences**

1. Egi M, Ogura H, Yatabe T, Atagi K, Inoue S, Iba T, et al. The Japanese Clinical Practice Guidelines for Management of Sepsis and Septic Shock 2020 (J-SSCG 2020). J Intensive Care. 2021 Aug 25;9(1):53. PMID: 34433491. doi: 10.1186/s40560-021-00555-7.

2. Gotschall T. EndNote 20 desktop version. J Med Libr Assoc. 2021 Jul 1;109(3):520-2. PMID: 34629985. doi: 10.5195/jmla.2021.1260.

3. Oami T, Okada Y, Sakuraya M, Fukuda T, Shime N, Nakada TA. Efficiency and workload reduction of semi-automated citation screening software for creating clinical practice guidelines: a prospective observational study. J Epidemiol. 2023 Dec 16. PMID: 38105001. doi: 10.2188/jea.JE20230227.

4. Ouzzani M, Hammady H, Fedorowicz Z, Elmagarmid A. Rayyan-a web and mobile app for systematic reviews. Syst Rev. 2016 Dec 5;5(1):210. PMID: 27919275. doi: 10.1186/s13643-016-0384-4.

5. Oami T, Okada Y, Nakada TA. Performance of a Large Language Model in Screening Citations. JAMA Netw Open. 2024 Jul 1;7(7):e2420496. PMID: 38976267. doi: 10.1001/jamanetworkopen.2024.20496.
